# Zinc Uptake, Translocation, and Remobilization in Winter Wheat as Affected by Soil Application of Zn Fertilizer

**DOI:** 10.3389/fpls.2019.00426

**Published:** 2019-04-16

**Authors:** Dun-Yi Liu, Yu-Min Liu, Wei Zhang, Xin-Ping Chen, Chun-Qin Zou

**Affiliations:** ^1^Key Laboratory of Plant-Soil Interactions, Ministry of Education, Center for Resources, Environment and Food Security, China Agricultural University, Beijing, China; ^2^Chongqing Key Laboratory of Efficient Utilization of Soil and Fertilizer Resources, College of Resources and Environment, Southwest University, Chongqing, China; ^3^Academy of Agricultural Sciences, Southwest University, Chongqing, China

**Keywords:** soil Zn application, wheat root morphology, Zn uptake, Zn translocation, Zn remobilization

## Abstract

Effect of zinc (Zn) application to soil on root growth and Zn uptake and translocation in winter wheat are poorly understood. This study evaluated the effect of soil Zn fertilization (0, 2.3, 5.7, 11.4, 22.7, 34.1 kg of Zn ha^−1^) on root growth and distribution, crop Zn uptake, root-to-shoot translocation of Zn, and remobilization of Zn from shoot to grain. Results of this study revealed that Zn application ≤11.4 kg ha^−1^ significantly increased root dry weight, root length density, and root surface area within 0–30 cm soil depth and higher rates of Zn application caused slight decreases in these root parameters. Shoot biomass and shoot Zn accumulation increased as Zn application rate increased mainly because of improved matching of root growth and enhanced availability of Zn in the topsoil layer. Post-anthesis Zn uptake by shoot increased and translocation of Zn from root to shoot decreased as rate of Zn application increased. The degree to which Zn accumulation in grain resulted from pre-anthesis remobilization vs. post-anthesis shoot uptake depended on Zn availability in soil; post-anthesis shoot uptake dominated at DTPA-Zn concentrations >7.15 mg kg^−1^, and pre-anthesis remobilization dominated at lower soil Zn levels. In conclusion, Zn uptake, translocation and remobilization to grain were affected by root growth and its matching with the availability of soil Zn. The results suggest that soils similar to the study soil should be fertilized to 30 cm depth with about 11.4 kg ha^−1^ Zn in order to obtain high yield and grain Zn concentration of wheat.

## Introduction

Zinc (Zn) deficiency, which is a common micronutrient disorder in cereal plants, reduces crop yields and nutritional quality. About 50% of cereal crops are cultivated on soils with low Zn availability worldwide (Alloway, 2009). Low concentrations of Zn in cereal products can cause Zn-deficiency related health problems in humans, especially in developing countries where cereal crops play an important role in satisfying daily calorie intake. Biofortification is receiving increasing attention in response to this problem (Cakmak, 2008; Bouis and Welch, 2010). Among biofortification approaches, Zn fertilization represents a short-term and effective strategy (Cakmak, 2008). Even when new cultivars with a strong genetic capacity for Zn absorption are developed, such absorption greatly depends on the size of the available Zn pool in soil. Fertilization of soil with Zn, which is usually applied as ZnSO_4_ 7H_2_O, appears to be important for ensuring the success of biofortification (Cakmak, 2008).

The biofortification of the Zn content in wheat grain via soil fertilization involves many physiological steps including the uptake of Zn by root and the root-to-shoot translocation and remobilization of Zn (Palmgren et al., 2008; Erenoglu et al., 2011). A better understanding of these steps should increase our ability to enhance the Zn content of grain via genetic and agronomic biofortification.

Zinc uptake by root was the first step in the movement of Zn from soil to grain. In the field of agronomy, Zn uptake has usually been measured as the product of root Zn concentration and root dry weight (RDW). Rose et al. (2013) also suggested that root surface area (RSA) and root length density (RLD) should be increased so as to increase the capture of immobile Zn. Application of Zn fertilizer to soil could increase the concentration of DTPA-Zn (i.e., available Zn) in soil and then have an effect on root growth. Although the effects of Zn application on root morphology (such as RDW and RSA) have been reported many times, the results have been inconsistent. Dong et al. (1995), for example, reported that RDW increased for three wheat genotypes as Zn application increased above 0.1 mg Zn kg^−1^ soil. Yang et al. (2011) found that Zn deficiency increased root growth of wheat. According to Chen et al. (2009), root length (RL) and RSA of a Zn-efficient rice genotype were decreased under both Zn-sufficient and Zn-severely deficient conditions relative to a moderately deficient condition. Li et al. (2005) reported that RL, RSA, and root volume of the non-hyperaccumulating ecotype of *Sedum alfredii* decreased significantly, whereas those of the hyperaccumulating ecotype were not affected by increasing concentrations of Zn^2+^ in the growth medium. Moreover, the partitioning of assimilates between root and shoot under Zn deficiency can be quite different for plants growing in soil vs. nutrient solution (Rengel and Graham, 1995). All of these reports involved pot or hydroponic experiments. Whether results obtained in pots or hydroponic solutions reflect results obtained in the field is unclear because few studies have been conducted in the field.

Translocation of Zn from root to shoot following Zn uptake by root is affected by many factors. Xue et al. (2014) suggested that optimization of the nitrogen (N) supply promotes root-to-shoot Zn translocation in wheat. In a maize field experiment, Dwivedi et al. (1975) found that the root: shoot ratio of Zn concentration increased with P application. A radio-labeled Zn (^65^Zn) experiment showed that ^65^Zn translocation rates were substantially higher in plants precultured without a Zn supply than in those precultured with a sufficient Zn supply (Erenoglu et al., 2011), indicating that the Zn nutrition of plant affects Zn translocation to grain. Several studies have investigated the effect of Zn application on root-to-shoot Zn translocation in crops. For example, for rice grown in agar nutrient solution, Impa et al. (2013) reported higher percentage of Zn allocation to root in Zn-sufficient than Zn-deficient conditions, suggesting a reduction in root-to-shoot translocation with Zn application. Using solution culture experiment, Stomph et al. (2014) reported that root-to-shoot Zn translocation in rice was affected by timing of Zn application. To our knowledge, very few studies investigated the correlation between fertilizer Zn dosage and root-to-shoot Zn translocation efficiency under field conditions. It is clear that application of Zn fertilizer to soil improves the yield and Zn nutrition of crops, but the effect of such application on Zn translocation from root to shoot under field conditions is not clear.

Accumulation of Zn in grain depends on the remobilization of Zn from shoot and the continued shoot uptake of Zn during the grain-filling stage (Waters et al., 2009; Impa et al., 2013). There has been a long-lasting debate as to whether post-anthesis shoot uptake or remobilization from vegetative tissue is more responsible for Zn accumulation in grain. The contributions of these two sources to Zn accumulation in grain may depend on environmental conditions and on the nutritional status of the crop (Kutman et al., 2012). Xue et al. (2012) and Zhang et al. (2015), for example, reported that Zn remobilization is critical for the accumulation of Zn in grain because shoot Zn uptake during grain filling can be limited by soil stresses such as low availability of Zn in soil, high CaCO_3_ concentration, high soil pH, and drought. In hydroponic culture with an ample Zn supply, however, net remobilization was not even detected (Waters et al., 2009). Kutman et al. (2012) also concluded that remobilization is critical for accumulation of Zn in grain when environmental Zn availability was restricted during grain filling, but that remobilization was less important when shoot uptake continued during grain development. To our knowledge, the threshold of soil Zn availability that determines whether Zn remobilization or Zn uptake dominates has not been determined for any soil under field conditions.

The current field study investigated how levels of Zn application to soil affected Zn uptake, translocation, and remobilization by winter wheat. The specific objectives were to determine the effect of soil Zn fertilization on (1) root morphology (RDW, RLD, and RSA) and the distribution in the soil profile, (2) Zn uptake by root, and Zn translocation from root to shoot, (3) the contribution of Zn remobilization via pre-anthesis vs. post-anthesis shoot Zn uptake to grain Zn as affected by Zn application levels.

## Materials and Methods

### Field Location

A field experiment was conducted during 2013–2014 and 2014–2015 winter wheat season, as part of a long-term experiment initiated in 2009 at the Quzhou Experimental Station (36.9°N, 115.0°E) in China. At beginning of the experiment, initial soil DTPA-extractable Zn concentration was 0.45 mg kg^−1^. The soil was a Zn deficient soil and some characteristics are shown in Supplementary Table S1. A standard agro-meteorological station was used to record mean temperature and precipitation (Supplementary Figure S1). During the two wheat growing seasons, the mean temperature was 11.9 and 11.4°C, and the total precipitation was 207 and 112 mm during wheat growing season of 2013–2014 and 2014–2015, respectively.

### Experimental Design

Wheat (*Triticum aestivum* L. cv. Liangxing 99) was planted on October 10 and October 11 in 2013 and 2014, and harvest on June 8 and June 10 in 2014 and 2015, respectively. The seeding rate was 187.5 kg ha^−1^, and the row spacing was 25 cm. Each year, experimental treatments were 0, 2.3, 5.7, 11.4, 22.7, and 34.1 kg ha^−1^ of Zn (as 0, 10, 25, 50, 100, and 150 kg ha^−1^ of ZnSO_4_ 7H_2_O, respectively), applied before crop sowing. Each treatment was represented by four replicate plots, and the plots were arranged in a randomized block design. The area of each plot was 75 m^2^ (15 m × 5 m). Zinc fertilizer (in the form of aqueous solutions) and basal compound fertilizer (N–P_2_O_5_–K_2_O: 15–15–15; 75 kg ha^−1^) was sprayed on the soil surface in each plot before planting, and then mixed into the soil by disk-plowing. At the stem elongation stage (GS31), additional 150 kg N ha^−1^ (as urea) was supplied. Irrigation was applied before start of winter, at stem elongation stage, and at anthesis stage. No obvious water, weed, or pest problems were observed during the experiment.

### Sampling and Analysis

To determine Zn remobilization, entire wheat shoot were sampled from two 50 cm lengths of two adjacent rows at anthesis stage (i.e., GS 65). In 2013–2014, wheat root were also collected at anthesis to study the effect of soil Zn application on root morphology including root dry weight (RDW), root length density (RLD), root surface area (RSA), root Zn uptake, and Zn translocation from root to shoot. To collect root samples, two blocks of soil were processed in each plot as described by Xue et al. (2014). Each block of soil (0.045 m^3^) had the following dimensions: 15 cm length in a row × 50 cm width × 60 cm depth. Root and soil samples in four soil layers (0–10, 10–20, 20–30, and 30–60 cm) were extracted sequentially in these two blocks. One part of the root samples was washed with deionized water and kept at >20°C before an image was captured with an optical scanner (Epson V700, Japan) and analyzed with software (WinRHIZO PRO 2004b, Canada) to determine RLD and RSA. The second block was used to measure RDW and root Zn concentration. To evaluate the effect of Zn fertilizer on wheat grain yield and Zn accumulation, samples were collected at crop maturity (i.e., at GS 92) every year. At maturity, entire wheat plants within 6 m^2^ (2 m × 3 m) random area of each experimental plot were harvested and separated into grains and straw manually. In this study, grain and straw yields were expressed on dry matter basis. All shoot samples and part of the root samples were rapidly washed with deionized water and then dried at 65°C in a forced-draft oven to constant weight. The plant samples were ground with a stainless steel grinder (RT-02A, Rong Tsong Precision Technology, Taiwan) for nutrient analysis. In addition to sampling four soil layers (0–10, 10–20, 20–30, and 30–60 cm) at anthesis stage in 2013–2014 cropping season, we also sampled top soil (0–30 cm depth) from each plot by using a stainless steel auger in an “S” route pattern at crop maturity in 2013–2014 and 2014–2015 cropping seasons. All soil samples were air-dried and crushed to pass 1 mm sieve. Soil Zn concentration was determined by end to end shaking (180 rpm) for 2 h of 10 g of soil with 20 ml of 0.005 mol L^−1^ DTPA (diethylene triamine pentacetic acid), 0.01 mol L^−1^ CaCl_2_, 0.1 mol L^−1^ TEA (tri-ethanol amine) buffered at pH 7.3 (Lindsay and Norvell, 1978). Zinc concentrations in the extractants were determined by ICP-OES (OPTIMA 7300 DV, PerkinElmer, United States). It has to be mentioned that the soil DTPA-Zn presented here was resulted from the consecutive application of Zn fertilizer over the last 10 growing seasons (from 2009 to 2014 in a wheat-maize rotation system), instead of only a single application.

### Calculations and Statistical Analysis

The term “shoot” refers to all aboveground parts of wheat plants. And the formula of main calculations are listed as following:

$$\begin{array}{c} \mathrm{Zinc accumulations in straw and root}\\ \;=\mathrm{Zn concentration in straw and root}\times \mathrm{its dry weight} \end{array}$$

$$\mathrm{Zinc harvest index}(\mathrm{ZnHI})=\frac{\mathrm{Grain Zn accumulation}}{\mathrm{Shoot Zn accumulation at maturity}}$$

$$\mathrm{Root length density}(\mathrm{RLD})=\frac{\mathrm{Root length}}{\mathrm{Specific soil volume}}$$

Supply capacity of Zn (SCZn) in each soil layer was calculated based on Zhang et al. (2013):

$$\mathrm{SCZn}=\sum \left(\mathrm{DTPA}-{\mathrm{Zn}}_{x}\times {\mathrm{RSA}}_{x}\right)$$

Pre-anthesis Zn uptake=shoot Zn accumulation at anthesis

$$\begin{array}{c} \mathrm{Post-anthesis shoot Zn uptake}\\ \;=\mathrm{shoot Zn accumulation at maturity}\\ \;-\mathrm{shoot Zn accumulation at anthesis}. \end{array}$$

$$\begin{array}{c} \mathrm{Pre-anthesis}(\mathrm{or post-anthesis})\mathrm{Zn accumulation}(\%)\\ \;=\frac{\mathrm{shoot Zn accumulation at anthesis or post-anthesis}}{\mathrm{shoot Zn accumulation at maturity}}\times 100\% \end{array}$$

$$\begin{array}{c} \mathrm{Zinc remobilization to the grain}\\ \;=\mathrm{shoot Zn accumulation at anthesis}\\ \;-\mathrm{straw Zn accumulation at maturity}. \end{array}$$

$$\begin{array}{c} \mathrm{Zinc remobilization efficiency}\\ \;=\frac{\mathrm{Zn remobilization}}{\mathrm{shoot Zn accumulation at anthesis}} \end{array}$$

$$\begin{array}{c} \mathrm{Share of grain Zn provided by remobilization}\\ \;=\frac{\mathrm{Zn remobilization to the grain}}{\mathrm{grain Zn accumulation}}\times 100\% \end{array}$$

$$\begin{array}{c} \mathrm{Share of post-anthesis shoot Zn uptake}\\ =\frac{\mathrm{grain Zn accumulation}-\mathrm{Zn remobilization to grain}}{\mathrm{grain Zn accumulation}}\times 100\%. \end{array}$$

Data normality and variance homogeneity were checked by SPSS 20.0 (IBM) before analysis. The effect of Zn application rate, year, and their interactions on the dependent variables was assessed by two-way repeated measures analysis of variance (ANOVA). In 2013–2014, the effects of Zn application rate on parameters associated with root was evaluated by one-way ANOVA. Means were separated by Duncan’s test at *P* < 0.05. SPSS was also used to assess the relationships between certain variables through Pearson’s correlation coefficients.

## Results

### Shoot Biomass and Zn Status of Wheat at Anthesis and Maturity Stages

At maturity stage in 2013–2014 cropping season, grain, and straw biomass of Zn treatment increased from 5.7 and 5.9 to 7.2 and 7.3 Mg ha^−1^, respectively (Table 1). Zinc concentration in grain and straw increased by 22.8–75.4 and 22.2–356.4% with different Zn application rates compared with no Zn application treatment, respectively. High as 58.6 and 53.4 mg kg^−1^ Zn concentration in grain and straw was observed in the 34.1 kg ha^−1^ Zn application treatment. Greater improvement of grain and straw accumulation was detected as affected by Zn application. Shoot biomass, Zn concentration and accumulation at anthesis in 2013–2014 were increased by Zn application (Table 2). In 2014–2015 cropping season, the same trend but different improvement degrees with Zn supply were detected (Tables 1, 2). The year also had significantly effects while the effect of interaction of Zn application × year was not significant (Tables 1, 2). ZnHI decreased significantly from 74 to 52 and 76 to 48 in the two cropping seasons, respectively, as the Zn application rate increased, while the effect of year and interaction of Zn application × year was not significant (Table 1).

**Table 1:** Biomass, Zn concentration, Zn accumulation of grain and straw at maturity stage and Zn harvest index (ZnHI) of winter wheat as affected by Zn application rate in two cropping seasons.

Cropping season	Zn rate (kg ha^−1^)	Biomass (Mg ha^−1^)		Zn concentration (mg kg^−1^)		Zn accumulation (g ha^−1^)		ZnHI
		Grain	Straw	Grain	Straw	Grain	Straw	
2013–2014	0	5.7 b	5.9 b	33.4 c	11.7 d	191 d	68 e	74 a
	2.3	6.0 b	6.2 ab	41.0 b	14.3 d	246 c	88 de	74 a
	5.7	5.9 b	5.8 b	43.3 b	23.2 c	251 c	132 d	66 b
	11.4	6.5 ab	6.7 ab	45.3 b	28.4 c	295 b	192 c	61 b
	22.7	7.1 a	7.0 ab	53.7 a	44.3 b	382 a	312 b	55 c
	34.1	7.2 a	7.3 a	58.6 a	53.4 a	417 a	388 a	52 c
2014–2015	0	7.3 b	7.7 b	32.8 d	9.7 d	237 d	76 c	76 a
	2.3	7.4 ab	7.9 ab	37.7 d	16.1 cd	280 cd	126 c	69 ab
	5.7	7.5 ab	7.6 b	44.6 c	22.5 c	337 c	171 c	67 bc
	11.4	7.8 ab	8.1 ab	51.6 b	34.9 b	399 b	284 b	59 c
	22.7	8.1 ab	8.2 ab	53.2 b	54.5 b	430 b	449 a	49 d
	34.1	8.1 a	8.5 a	61.3 a	64.4 a	495 a	541 a	48 d
Source of variation							
Zn		0.000	0.032	0.000	0.000	0.000	0.000	0.000
Year		0.001	0.000	0.358	0.017	0.002	0.001	0.113
Zn^∗^Year		0.555	0.924	0.178	0.171	0.204	0.056	0.416

*Values are average of four replications. For each cropping season, means in a column followed by same letters are not significant difference at P < 0.05 by Duncan’s multiple comparison test.*

**Table 2:** Biomass, Zn concentration, and Zn accumulation of shoot at anthesis stage of winter wheat as affected by Zn application rate in two cropping seasons.

Cropping season	Zn rate (kg ha^−1^)	Biomass (Mg ha^−1^)	Zn concentration (mg kg^−1^)	Zn accumulation (g ha^−1^)
2013–2014	0	7.8 b	24.4 d	190 c
	2.3	9.4 ab	25.8 cd	242 bc
	5.7	9.1 ab	29.7 cd	265 bc
	11.4	9.2 ab	34.7 c	321 b
	22.7	9.5 a	46.0 b	443 a
	34.1	8.9 ab	57.1 a	506 a
2014–2015	0	8.8 c	26.1 e	229 e
	2.3	9.2 bc	31.5 de	291 de
	5.7	9.5 abc	36.0 d	339 d
	11.4	10.5 a	43.0 c	453 c
	22.7	10.5 ab	55.1 b	573 b
	34.1	10.7 a	63.1 a	672 a
Source of variation				
Zn		0.008	0.005	0.000
Year		0.002	0.007	0.001
Zn^∗^Year		0.371	0.435	0.324

*Values are average of four replications. For each cropping season, means in a column followed by same letters are not significant difference at P < 0.05 by Duncan’s multiple comparison test.*

### Effect of Zn Fertilization on Available Soil Zn

**FIGURE 1:**
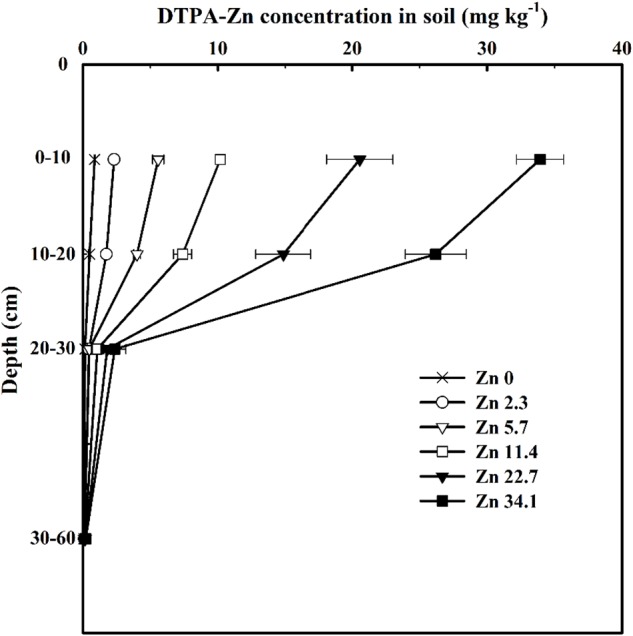
Soil DTPA-Zn concentrations in different soil layers at the anthesis stage of winter wheat as affected by Zn application rate in the 2013–2014 cropping season. Values here were resulted from the consecutive application of Zn fertilizer over the last 10 growing seasons, instead of only a single addition. Values are average ± SE of four replications.

In 2013–2014, available soil Zn (DTPA-Zn) concentrations decreased with soil depth in all six treatments and progressively increased at any soil depth as the quantity of Zn applied increased (Figure 1). Soil DTPA-Zn concentration range from 0.89 to 33.93 mg kg^−1^ in 0–10 cm, 0.49 to 26.19 mg kg^−1^ in 10–20 cm, 0.15 to 2.37 mg kg^−1^ in 20–30 cm, and 0.09 to 0.20 mg kg^−1^ in 30–60 cm soil layer among treatments.

### RDW, RLD, RSA, and Root Zn Status at Each Soil Depth

Root dry weight decreased with soil depth irrespective of Zn application rate. About 85% of the root biomass was in the 0–30 cm soil layer (Table 3). RDW at 0–30 cm depths significantly increased from 483 to 700 g ha^−1^ as the Zn application rate increased from 0 to 11.4 kg ha^−1^ Zn but slightly decreased with further increases in the application rate, especially in the upper two soil layers (0–10 cm and 0–20 cm). However, the RDW was still higher in the 22.7 and 34.1 kg ha^−1^ Zn treatments than in the 0 kg ha^−1^ Zn treatment. RDW at 30–60 cm soil depth was not significantly affected by Zn application rate (Table 3). The effects of Zn fertilization on RLD and RSA and their distribution in the soil profile were similar to the effects of Zn fertilization on RDW (Table 3).

**Table 3:** Root dry weight (RDW), root length density (RLD), and root surface area (RSA) of winter wheat at the anthesis stage as affected by Zn application rate and soil depth in the 2013–2014 cropping season.

Parameter	Zn rate (kg ha^−1^)	Soil depth (cm)					
		0–10	10–20	20–30	0–30	30–60	0–60
RDW (kg ha^−1^)	0	260 b	155 b	68 b	483 b	80 a	563 b
	2.3	317 ab	211 ab	102 ab	630 a	100 a	730 a
	5.7	337 ab	236 a	83 b	656 a	100 a	756 a
	11.4	373 a	216 ab	111 ab	700 a	115 a	815 a
	22.7	330 ab	217 ab	135 a	682 a	96 a	778 a
	34.1	298 b	178 b	114 ab	590 ab	95 a	685 ab
RLD (cm cm^−3^)	0	1.33 b	1.84 b	1.02 a	1.40 b	0.28 b	0.84 b
	2.3	1.47 b	2.36 ab	1.41 a	1.75 ab	0.39 ab	1.07 ab
	5.7	1.77 ab	2.93 ab	1.40 a	2.03 a	0.48 a	1.25 ab
	11.4	2.10 a	3.32 a	1.59 a	2.34 a	0.46 a	1.40 a
	22.7	1.80 ab	3.08 ab	1.50 a	2.13 a	0.361 ab	1.24 ab
	34.1	1.49 ab	2.96 ab	1.58 a	2.01 a	0.43 a	1.22 ab
RSA (cm^2^ cm^−2^)	0	1.02 b	1.19 b	0.75 b	2.95 c	0.68 b	3.63 c
	2.3	1.04 b	1.44 ab	0.99 a	3.46 bc	0.87 ab	4.33 bc
	5.7	1.32 ab	1.88 ab	1.10 a	4.30 ab	1.08 a	5.38 ab
	11.4	1.64 a	2.09 a	1.18 a	4.92 a	1.04 a	5.96 a
	22.7	1.32 ab	2.06 a	1.13 a	4.51 ab	0.90 ab	5.40 ab
	34.1	1.11 b	1.89 ab	1.36 a	4.37 ab	1.06 ab	5.43 ab

*Values are average of four replications. Means in a column followed by same letters are not significant difference at P < 0.05 by Duncan’s multiple comparison test.*

Zinc concentration and accumulation in root increased significantly with Zn application rate irrespective of soil depth, and the effect of application rate was greater at 0–30 cm depth than at 30–60 cm depth (Table 4). Zinc concentrations and accumulation in root were higher in the upper 30 cm of soil than in the lower 30 cm of soil; root Zn concentration was highest at 10–20 cm soil depth, and root Zn accumulation was highest at 0–10 cm soil depth (Table 4). More than 90% of the Zn that accumulated in root was in the upper 30 cm of soil (Table 4). The supply capacity of Zn (SCZn) at the anthesis stage increased significantly in each of soil layer as the Zn application rate increased (Table 4).

**Table 4:** Zinc concentrations and accumulations in root of winter wheat and the supply capacity of Zn (SCZn) at the anthesis stage as affected by Zn application rate and soil depth in the 2013–2014 cropping season.

Parameter	Zn rate (kg ha^−1^)	Soil depth (cm)					
		0–10	10–20	20–30	0–30	30–60	0–60
Zn concentration in root (mg kg^−1^)	0	29.5 e	36.3 d	20.4 d	30.5 d	16.7 b	28.6 d
	2.3	34.3 de	40.6 d	24.0 c	34.6 c	17.3 b	32.4 d
	5.7	47.1 cd	58.6 c	30.4 cd	49.5 c	18.1 ab	45.3 c
	11.4	61.6 c	74.6 c	37.9 bc	62.3 c	18.4 ab	56.1 c
	22.7	115.4 b	127.4 b	40.7 b	105.7 b	18.3 ab	94.9 b
	34.1	153.0 a	178.3 a	55.6 a	141.7 a	19.4 a	124.6 a
Zn accumulation in root (g ha^−1^)	0	7.7 c	5.6 d	1.4 c	14.7 d	1.4 b	16.1 d
	2.3	10.7 c	8.6 cd	2.7 bc	22.0 cd	1.7 ab	23.7 cd
	5.7	15.9 bc	14.2 bc	2.6 bc	32.7 bc	1.8 ab	34.5 bc
	11.4	23.2 b	16.1 b	4.3 ab	43.6 b	2.2 a	45.8 b
	22.7	38.7 a	27.9 a	5.6 a	72.2 a	1.7 ab	73.9 a
	34.1	45.4 a	31.9 a	6.3 a	83.6 a	1.8 ab	85.4 a
Zn accumulation in root as a function of depth (%)	0	47.6 a	34.7 a	9.3 a	91.6 d	8.4 a	100
	2.3	44.2 a	37.3 a	10.8 a	92.3 cd	7.7 ab	100
	5.7	46.8 a	39.8 a	7.9 a	94.5 bc	5.5 bc	100
	11.4	50.3 a	35.7 a	9.3 a	95.3 ab	4.7 cd	100
	22.7	51.2 a	38.2 a	8.2 a	97.6 a	2.4 d	100
	34.1	53.1 a	37.3 a	7.5 a	97.9 a	2.1 d	100
^*a*^SCZn in the soil profile (cm^2^ cm^−2^ × mg kg^−1^)^*a*^	0	0.88 d	0.60 d	0.12 b	1.60 d	0.06 c	1.66 d
	2.3	2.35 d	2.50 cd	0.52 b	5.37 d	0.10 bc	5.46 d
	5.7	7.48 cd	7.43 cd	0.55 b	15.45 cd	0.12 abc	15.57 cd
	11.4	16.67 c	15.40 c	1.27 b	33.35 c	0.17 ab	33.52 c
	22.7	27.22 b	32.00 b	2.45 ab	61.67 b	0.15 abc	61.82 b
	34.1	38.16 a	48.31 a	4.15 a	90.63 a	0.22 a	90.85 a

*^a^SCZn = DTPA-Zn_x_ × RSA_x_, where DTPA-Zn_x_ is the soil DTPA-Zn concentration and RSA_x_ is the root surface area in soil layer x. Values are average of four replications. Means in a column followed by same letters are not significant difference at P < 0.05 by Duncan’s multiple comparison test.*

### Relationship Between Zn Accumulation in Root and Shoot

**FIGURE 2:**
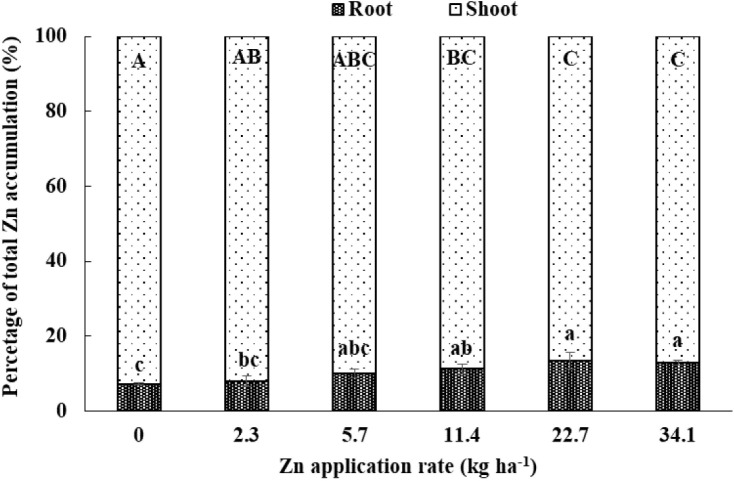
Zinc accumulation in root and shoot as a percentage of total (root + shoot) Zn accumulation at the anthesis stage of winter wheat as affected by Zn application rate in the 2013–2014 cropping season. Values are average + SE of four replications. Same lowercase letters indicate no significant differences (*P* < 0.05) among treatments for root Zn accumulation relative to total Zn accumulation. Same uppercase letters indicate no significant differences (*P* < 0.05) among treatments for shoot Zn accumulation relative to total Zn accumulation.

As the Zn application rate increased, total Zn accumulation (including root and shoot) significantly increased (Tables 2, 4). The percentage of Zn accumulated in root relative to total Zn accumulation significantly increased as the Zn application rate increased, while the percentage of Zn accumulated in shoot relative to total Zn accumulation significantly decreased as the Zn application rate increased (Figure 2). Without Zn application, Zn accumulation in root and shoot was 16.1 and 190 g ha^−1^, while 5.3 times in root and 2.7 times in shoot of Zn accumulation in the highest Zn application treatment. Root Zn accumulation relative to the total Zn accumulation increased from 7.2 to 12.9% and shoot Zn accumulation relative to the total Zn accumulation decreased from 92.8 to 87.1% with the increasing Zn rate. Shoot Zn accumulation at anthesis and maturity stages and grain Zn concentration were positively correlated with the root Zn concentration, soil DTPA-Zn concentration, and SCZn, especially at 0–30 cm soil depth (Table 5).

**Table 5:** Pearson correlation coefficients among shoot Zn accumulation at anthesis stage (SZnA-A), grain Zn concentration (GZnC), shoot Zn accumulation at maturity stage (SZnA-M), and root Zn concentration (RZnC), root dry matter (RDW), root surface area (RSA), soil DTPA-Zn, supply capacity of Zn (SCZn) in 0–30 and 30–60 cm soil depths at anthesis.

	Soil depth (cm)	SZnA-A	GZnC	SZnA-M
RZnC	0–30	0.855^∗∗^	0.896^∗∗^	0.933^∗∗^
	30–60	0.345^ns^	0.471^∗^	0.486^∗^
RDW	0–30	0.153^ns^	0.333^ns^	0.222^ns^
	30–60	0.269^ns^	0.126^ns^	0.047^ns^
RSA	0–30	0.442^∗^	0.446^∗^	0.415^∗^
	30–60	0.400^ns^	0.340^ns^	0.262^ns^
DTPA-Zn	0–30	0.908^∗∗^	0.873^∗∗^	0.906^∗∗^
	30–60	0.344^ns^	0.502^∗^	0.623^∗∗^
SCZn	0–30	0.891^∗∗^	0.849^∗∗^	0.881^∗∗^
	30–60	0.421^∗^	0.507^∗^	0.583^∗∗^

*^∗^, ^∗∗^ indicate significant difference at P < 0.05 and <0.01, respectively. ns indicates non-significance.*

### Shoot Zn Uptake and Zn Remobilization During the Grain-Filling Period

The shoot Zn accumulation at maturity (i.e., pre-anthesis + post-anthesis) and post-anthesis shoot Zn accumulation significantly increased with Zn application rate. In contrast, the pre-anthesis Zn accumulation and Zn remobilization efficiency decreased with Zn application rate (Table 6). Share of grain Zn provided by remobilization decreased from 65 to 28% while share of post-anthesis shoot Zn uptake increased from 35 to 72% with the increasing Zn application. Zinc remobilization to the grain was unaffected by Zn application (Table 6). The year and the interaction of Zn application × year was not significant except the effect of year for the shoot Zn accumulation at maturity (Table 6).

**Table 6:** Zinc parameters of winter wheat during the 2013–2014 and 2014–2015 cropping seasons as affected by Zn application rate.

Cropping season	Zn rate (kg ha^−1^)	Shoot Zn accumulation at maturity(g ha^−1^)	Zn accumulation ratio		Zn remobilization to grain(g ha^−1^)	Zn remobilization efficiency(%)	Share of grain Zn provided by remobilization (%)	Share of post-anthesis shoot Zn uptake (%)
			Pre-anthesis (%)	Post-anthesis (%)				
2013–2014	0	259 e	74 a	26 b	122 a	48 a	65 a	35 b
	2.3	335 d	72 a	28 b	153 a	46 a	62 a	38 b
	5.7	383 d	70 ab	30 ab	133 a	35 ab	53 ab	47 ab
	11.4	487 c	67 ab	33 ab	129 a	28 abc	46 ab	54 ab
	22.7	694 b	65 b	35 a	131 a	30 bc	34 ab	66 ab
	34.1	805 a	64 b	36 a	118 a	16 c	28 b	72 a
2014–2015	0	313 e	73 a	27 b	153 a	49 a	65 a	35 b
	2.3	406 de	72 a	28 b	165 a	41 ab	59 a	41 b
	5.7	508 d	67 ab	33 ab	168 a	34 b	50 ab	50 ab
	11.4	683 c	66 ab	34 ab	170 a	26 bc	43 ab	57 ab
	22.7	879 b	65 ab	35 ab	124 a	16 c	32 b	68 a
	34.1	1036 a	65 b	35 a	132 a	13 c	27 b	73 a
Source of variation								
Zn rate (Zn)		0.000	0.028	0.028	0.462	0.000	0.003	0.003
Year (Y)		0.000	0.883	0.883	0.445	0.177	0.716	0.716
Zn × Y		0.143	0.907	0.907	0.686	0.532	0.854	0.854

*Values are average of four replications. For each cropping season, means in a column followed by same letters are not significant difference at P < 0.05 by Duncan’s multiple comparison test.*

**FIGURE 3:**
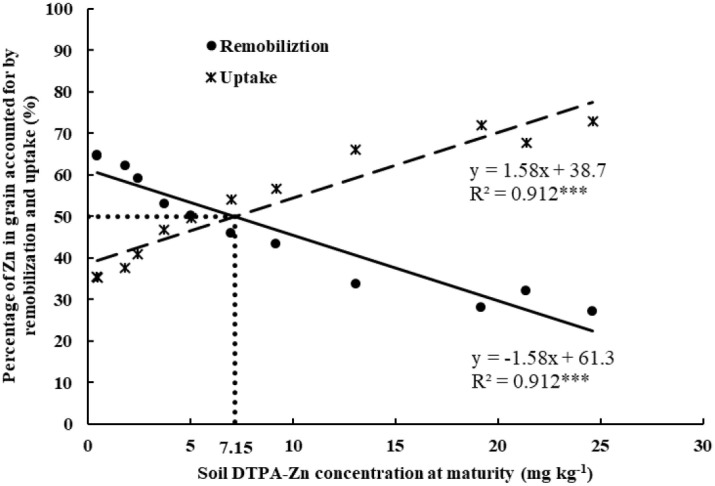
Relationship between soil DTPA-Zn concentration at maturity and share of wheat grain Zn provided by remobilization of pre-anthesis (%) and post-anthesis shoot uptake (%) in 2013–2014 and 2014–2015 cropping seasons. The linear regressions are significant difference at *P* < 0.001. The dotted vertical line indicates the soil DTPA-Zn concentration at which Zn remobilization and Zn uptake contribute equally to grain Zn content.

The relationship between soil DTPA-Zn concentration at maturity and the percentage of wheat grain Zn provided by pre-anthesis remobilization vs. post-anthesis shoot Zn uptake showed that uptake was more important than remobilization when DTPA-Zn values were greater than 7.15 mg kg^−1^ and that remobilization was more important than uptake when DTPA-Zn values were less than 7.15 mg kg^−1^ (Figure 3).

## Discussion

### Moderate Zn Application Enhanced Root Morphology of Wheat

In our field experiment, wheat biomass at anthesis and maturity stage was significantly increased with Zn application, which was consistent with previous studies (Cakmak et al., 1999; Liu et al., 2017). Wheat root were mainly distributed in the upper soil layers, which was consistent with Dwyer et al. (1996). The soil in this study had an initial DTPA-Zn concentration of only 0.45 mg kg^−1^, indicating that it was Zn deficient and would limit the root growth of wheat (Chen et al., 2009). Zinc application at ≤50 kg ha^−1^ significantly increased RDW, RLD, and RSA in the 0–30 cm soil layer but higher rates caused slight decreases in these parameters. The slight reductions in RDW, RLD, and RSA with Zn application rate >11.4 kg ha^−1^ were probably due to the very high levels of available Zn in the upper soil layers (0–20 cm) (Table 4). The soil DTPA-Zn in the upper soil layers (0–20 cm) in the 22.7 and 34.1 kg ha^−1^ Zn treatments was 17.8 mg kg^−1^ and 30.1 mg kg^−1^, respectively, which was significantly higher than the Zn biofortification “target value” of 4.09 mg kg^−1^ for wheat (Liu et al., 2017). Extremely high levels of available Zn in soil may inhibit root elongation (Godbold et al., 1983). In the current study, the high concentrations of available Zn in soil might have acted as a signal that was perceived by root and that resulted in decreases in RDW, RLD, and RSA. On the other hand, root morphological traits were better when Zn was applied at high rate (22.7 and 34.1 kg ha^−1^ Zn) than when Zn was not applied at all. The rapid increase in root Zn concentration after Zn application indicated that Zn uptake by root increased as Zn application increased even though the high rates caused slight decreases in RDW, RLD, and RSA.

### Matching of Root and Available Zn in Topsoil Layer Dominated Zn Accumulation

Because root biomass and soil DTPA-Zn concentrations were much greater in the upper than in lower soil layers, more than 90% of the total Zn was detected in the 0–30 cm soil layer, and the quantity in this layer increased with Zn application rate. This distribution of root and available soil Zn resulted in differences in shoot Zn uptake. The parameter SCZn, which was introduced to quantify the spatial matching between RSA and available soil Zn, was previously found to be the best predictor of Zn accumulation in shoot in pot experiments (Zhang et al., 2013; Liu et al., 2017). In the current study, SCZn also increased with the Zn application rate, especially in the upper soil layer. Consistent with the results of previous pot experiments (Zhang et al., 2013; Liu et al., 2017), the current results indicate that the matching of available soil Zn and root is critical for shoot Zn uptake in the field. The increase in Zn uptake protected wheat from Zn deficiency, which resulted in increased shoot biomass of wheat. The status of Zn in the wheat tissue was most strongly correlated with SCZn in the 0–30 cm soil layer. These results indicate that Zn fertilizer should applied to the upper 0–30 cm of soil.

Shoot biomass and Zn accumulation were also affected by the source of variation “year” (Tables 1, 2, 6). The reasons might be the different climate conditions such as precipitations and mean temperatures during the two wheat growing seasons (Supplementary Table S1). There was more precipitations in May, 2015, which could alleviate the stress of heat and drought in milking stage of wheat. In addition, Zn have a cumulative fertilizer effect (Grewal and Graham, 1998). Our previous study also showed that the overall response of soil DTPA-Zn to the application of Zn fertilizer presented an increasing trend with each successive year (Liu et al., 2017). The different available soil Zn between two cropping seasons might have an effect on shoot Zn uptake and biomass.

### Zinc Application Decreased Zn Translocation

The percentage of Zn translocated from root to shoot decreased as the Zn application rate increased, which was consistent with results reported by Erenoglu et al. (2011). With high Zn availability at the soil-root interface, substantial symplastic Zn fluxes may occur, and the delivery of Zn to the root xylem exclusively via symplast is kinetically challenging (White et al., 2002). In addition, the expression of some genes related to xylem loading and unloading of Zn, like those that encode heavy metal ATPase and yellow stripe-like (YSL) transporters, was suppressed by high levels of available Zn, perhaps because of competition for recognition sites (Curie et al., 2009; Palmer and Guerinot, 2009).

### Zinc Accumulation in Grains via Pre-anthesis Remobilization vs. Post-anthesis Shoot Uptake Depended on Available Soil Zn

Pre- and post-anthesis Zn uptake in shoot were significantly affected by Zn application in the current study. Post-anthesis Zn accumulation in shoot increased as available soil Zn increased, which was consistent with studies on N (Ercoli et al., 2008; Meng et al., 2013). The Zn in grain has two sources: (1) recently acquired Zn that is transferred directly (via translocation from root without intermediate storage in shoot) to kernels during the grain-filling stage, and (2) the less-recently acquired Zn that has been stored in shoot and that is subsequently transferred (remobilized) to grain. The first source reflects post-anthesis shoot uptake while the second source reflect pre-anthesis remobilization. When Zn supply is restricted by soil or weather conditions, plants have difficulty in acquiring Zn, post-anthesis shoot Zn uptake can be limited, and the Zn in grain mostly depends on the remobilization of Zn from vegetative parts (Xue et al., 2012). Under Zn-sufficient conditions, however, shoot uptake of Zn during grain-filling is the main source of Zn in grain (Impa et al., 2013). As indicated by (Kutman et al., 2012) and the results of the current study (Table 6), the degree to which Zn accumulation in grains resulted from pre-anthesis remobilization vs. post-anthesis shoot uptake depends on the availability of soil Zn. In the current study, the major source of Zn in grain depended on the concentration of available soil Zn (DTPA-Zn), and the critical concentration for the study soil was 7.15 mg of DTPA-Zn kg^−1^. At higher values, post-anthesis shoot uptake was more important than remobilization, and the opposite was true at lower values. To our knowledge, this is the first time that this value has been determined under field conditions. Recognition of this value in different soils will help guide Zn management in wheat production.

## Conclusion

In this study, RDW, RLD, and RSA were increased by moderate rates of Zn application but were slightly reduced by excessive rates. The closer spatial matching of root and available Zn in the upper vs. the lower layers of soil resulted in much greater Zn uptake in the upper layers. The percentage of Zn translocated from root to shoot was decreased while post-anthesis shoot Zn accumulation was increased by Zn application. The contributions of Zn remobilization from pre-anthesis and Zn uptake during post-anthesis to the accumulation of Zn in grain depended on the concentration of available soil Zn (DTPA-Zn); the critical value that determined whether remobilization or uptake was more important was 7.15 mg kg^−1^ in the study soil. The results presented here indicate that wheat planted in soil similar to the study soil should be fertilized with 5.7–11.4 kg ha^−1^ Zn, and that fertilizer should be applied to the 0–30 cm soil layer. These results have increased our understanding of Zn uptake and translocation in wheat and should be useful for guiding Zn management so as to achieve higher yields and higher grain Zn contents.

## Author Contributions

X-PC and C-QZ conceived and designed the experiments. D-YL, Y-ML, and WZ performed the experiments. D-YL and C-QZ analyzed the data and wrote the manuscript.

## Conflict of Interest Statement

The authors declare that the research was conducted in the absence of any commercial or financial relationships that could be construed as a potential conflict of interest.
