# Supplementary figures and images for: Zinc Uptake, Translocation, and Remobilization in Winter Wheat as Affected by Soil Application of Zn Fertilizer
Source: Front Plant Sci. 2019 Apr 16;10:426. doi: 10.3389/fpls.2019.00426 (PMC6477674; doi:10.3389/fpls.2019.00426)

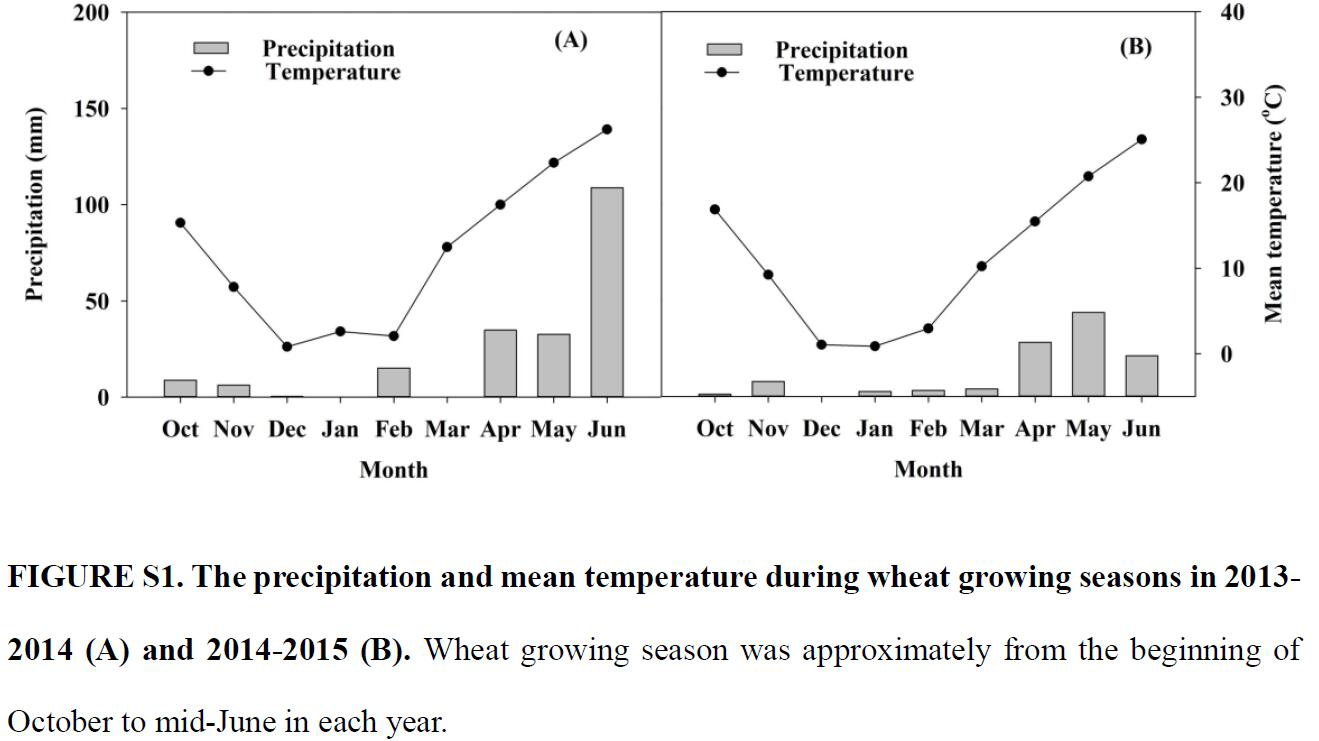

Supplement: Supplementary file 1 [file Image_1.tif]
