# Supplementary material for: Zinc Uptake, Translocation, and Remobilization in Winter Wheat as Affected by Soil Application of Zn Fertilizer
Source: Front Plant Sci. 2019 Apr 16;10:426. doi: 10.3389/fpls.2019.00426 (PMC6477674; doi:10.3389/fpls.2019.00426)
Supplement: Supplementary file 2 [file Table_1.DOCX]

**TABLE S1. Soil characteristics of the field experiment.**

| **Parameters** | **Descriptions** |
| --- | --- |
| Soil texture | silt loam |
| Clay <2 μm (%) | 7.9 |
| Silt 2–20 μm (%) | 55.3 |
| Sand 20–2000 μm (%) | 36.8 |
| Cation exchange capacity (CEC) (cmol kg^-1^) | 11.6 |
| Organic matter concentration (g kg^-1^) | 12.1 |
| Total N concentration (g kg^-1^) | 0.62 |
| Olsen-P (mg kg^-1^) | 6.9 |
| pH | 8.0 |
| DTPA-Zn concentration (mg kg^-1^) | 0.45 |
| CaCO_3_ concentration (%) | 4.5 |
